# Histamine up‐regulates oncostatin M expression in human M1 macrophages

**DOI:** 10.1111/bph.14796

**Published:** 2019-12-11

**Authors:** Susanne Mommert, Marius Hüer, Katrin Schaper‐Gerhardt, Ralf Gutzmer, Thomas Werfel

**Affiliations:** ^1^ Division of Immunodermatology and Allergy Research, Department of Dermatology and Allergy Hannover Medical School Hannover Germany

## Abstract

**Background and Purpose:**

Human monocyte‐derived M1 macrophages develop in relation to growth factors, bacterial products, and cytokines in a local micro‐environment. M1 macrophages produce pro‐inflammatory mediators, in particular, oncostatin M (OSM), which is secreted from the cells in response to the active complement component C5a. As C5a also releases histamine from human mast cells and shows immune modulatory functions similar to histamine in regulating expression of the IL‐12 cytokine family, we investigated the effects of histamine on OSM expression in human M1 macrophages.

**Experimental Approach:**

Cytokine expression was analysed by real‐time quantitative PCR and elisa techniques. Normal human epidermal keratinocytes were stimulated with supernatants from activated M1 macrophages, and phosphorylation of STAT3 was assessed by flow cytometry.

**Key Results:**

OSM mRNA expression was highly up‐regulated by histamine and agonists targeting the histamine H_1_ H_2_, and H_4_ receptors in human M1 macrophages and by C5a, which was used as control stimulus. Protein levels of OSM and IL‐6 were up‐regulated by histamine. Supernatants from histamine‐stimulated, fully differentiated M1 macrophages were able to phosphorylate STAT3 in normal human epidermal keratinocytes.

**Conclusions and Implications:**

The up‐regulation of OSM expression in response to histamine and C5a shown in this study provides further evidence that histamine and C5a, acting through their GPCRs, have almost equal functional effects in cells of the monocyte lineage. Both mediators OSM and IL‐6 have the capability to activate human keratinocytes. This effect may have an influence on the course of inflammatory skin diseases.

**Linked Articles:**

This article is part of a themed section on New Uses for 21st Century. To view the other articles in this section visit http://onlinelibrary.wiley.com/doi/10.1111/bph.v177.3/issuetoc

**Abbreviations**
NHEKs: normal human epidermal keratinocytes
OSM: oncostatin M

**What is already known**

- Stimulation of C5a receptors or histamine receptors reduces TLR‐induced up‐regulation of the IL‐12 family cytokines.
- C5a up‐regulates oncostatin M expression in human M1 macrophages.

**What this study adds**

- Histamine also up‐regulates expression of oncostatin M and thereby may activate human keratinocytes.

**What is the clinical significance**

- Elevation of oncostatin M levels by histamine may critically regulate pathological skin immune responses.
- These findings may help to identify novel approaches to treatments for human inflammatory skin diseases.

## 1 INTRODUCTION

Human macrophages in skin or in different tissues derive from resident or circulating monocytes, which differentiate according to the micro‐environment at their local niche either in more pro‐inflammatory, classically activated M1 macrophages or in more anti‐inflammatory alternatively activated M2 macrophages. Beyond the expression of characteristic surface markers, the subtypes differ in gene expression of M1‐ or M2‐specific chemokines and cytokines. For example, M1 macrophages are characterized by the release of the chemokines CXCL10, CXCL11 and CCL5 and the cytokines IL‐12, TNF‐α, IL‐1β and oncostatin M (OSM) (Biswas & Mantovani, 2010; Kastl et al., 2008; Mosser & Edwards, 2008).

The cytokine OSM was discovered in supernatants of the human monocytic cell line U937 activated with phorbol myristate acetate (Zarling et al. 1986) and was later added to the family of gp130 (or IL‐6/LIF) cytokines (Richards, 2013). Human OSM is able to bind to two different receptor complexes: The type I complex consisting of gp130 and the leukaemia inhibitory factor receptor (LIFR; gp130/LIFRb) or the type II receptor complex consisting of gp130 and the OSM receptor (gp130/OSMRB) (Hermanns, 2015). Besides its role in tumour progression (Sterbova, Karlsson, & Persson, 2018), bone marrow inflammation (Guihard et al., 2015; Torossian et al., 2017), CNS inflammatory diseases (Glezer & Rivest, 2010), and in human airway diseases (Fritz et al., 2011; Mozaffarian et al., 2008), OSM expression is up‐regulated in inflammatory skin conditions such as in psoriatic or in atopic dermatitis skin (Finelt, Gazel, Gorelick, & Blumenberg, 2005; Guilloteau et al., 2010). In vitro stimulation of normal human epidermal keratinocytes (NHEKs) with OSM in combination with other pro‐inflammatory cytokines showed a transcriptional profile of antimicrobial peptides and of chemokine expressions that resembles in part that of lesional psoriatic skin (Guilloteau et al., 2010). OSM alone also regulates mRNA transcripts of antimicrobial peptides, in particular the expression of S100A7 and human beta defensin 2 (also defensin 4A) in NHEKs (Boniface et al., 2007; Guilloteau et al., 2010).

OSM production is increased in human dendritic cells or macrophages by lipopolysaccharide (LPS), fixed *Staphylococcus aureus*, or after stimulation by PGE_2_ respectively (Repovic & Benveniste, 2002; Suda et al., 2002). Up‐regulation of OSM expression in response to the potent anaphylatoxin C5a, which is generated during complement activation through cleavage of C5, had also been described for human macrophages (Kastl et al., 2008).

In contrast, stimulation with C5a inhibits TLR4‐induced up‐regulation of IL‐12, IL‐23, and IL‐27 mRNA and protein expression in human macrophages and monocytes (Hawlisch et al., 2005; Wittmann et al., 1999). Surprisingly, the TLR4‐induced up‐regulation of IL‐27 expression as well as the TLR3‐induced expression of IL‐12 were also down‐regulated by histamine via the histamine H_2_ and H_4_ receptors in monocytes and antigen‐presenting cells (Gschwandtner et al., 2012; Gschwandtner, Schakel, Werfel, & Gutzmer, 2011; Gutzmer et al., 2005), pointing out that both C5a and histamine regulate expression of the IL‐12 family cytokines in a similar fashion.

Histamine is a pleiotropic mediator that is present in elevated concentrations in human skin in inflammatory skin diseases such as psoriasis (Krogstad, Lonnroth, Larson, & Wallin, 1997) or atopic dermatitis (Ruzicka & Gluck, 1983) mediating its effect through four G‐protein coupled receptors (Schaper‐Gerhardt et al., 2018).

The functional similarities between C5a and histamine, for example, showing a down‐regulation of Th1 promoting cytokines in inflammatory conditions, prompted us to investigate if histamine also increases OSM production in the same fashion as described for C5a in human M1 macrophages (Kastl et al., 2008).

## 2 METHODS

### 2.1 Isolation of monocyte derived macrophages

Residual blood samples from platelet apheresis disposables used for routine platelet collection and of regular anonymous healthy donors (buffy coats) served as source material for the isolation of human peripheral blood mononuclear cells (PBMCs).

The blood samples were randomly selected by the Transfusion Medicine MH‐Hannover fulfilling the criteria of a bias‐free representation of the population.

We have no access to individual data such as demographic or anamnestic characteristics that may influence the response to histamine or to the other histamine receptor agonists. Therefore, no inclusion/exclusion criteria of the human donors are defined in advance.

**Figure 1:**
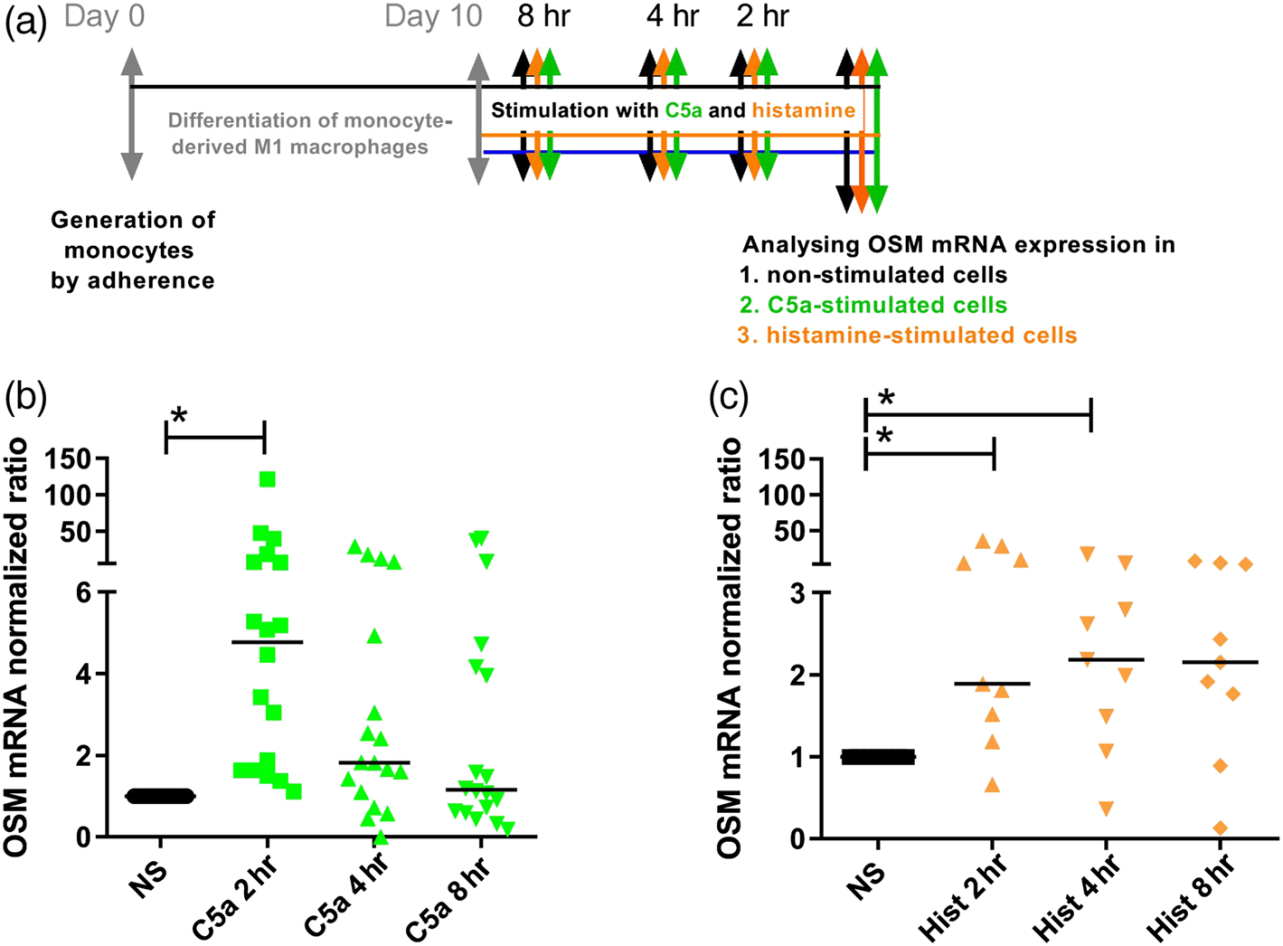
Histamine and C5a up‐regulate OSM mRNA expression with different time‐courses in monocyte‐derived human M1 macrophages. (a) Primary human monocytes were obtained from peripheral blood mononuclear cells after 2‐hr adherence. M1 macrophages were differentiated from primary human monocytes in the presence of GM‐CSF (10 ng·ml^−1^) for 10 days and then stimulated with C5a (10 ng·ml^−1^) or histamine (10 μM) for different time periods as indicated. (b) OSM mRNA expression after treatment for different time periods as indicated with C5a (*n* = 18 independent donors and experiments). (c) OSM mRNA expression after treatment for different time periods as indicated with histamine (Hist; *n* = 9 independent donors and experiments). The amount of the target mRNA relative to the amount of the reference gene, rps 20 mRNA in each stimulated sample was normalized to the amount of the target mRNA relative to the amount of the reference gene in the non‐stimulated sample (calibrator) and expressed as normalized ratio. This was calculated using the comparative Ct method also known as the ΔΔCt method provided by the Software LC 480 (Roche Molecular Biochemicals). Data shown are individual values with medians. *P < .05; significantly different as indicated; Friedman Dunn's Multiple Comparisons test selected pairs. Ct method, crossing point method; NS, non‐stimulated; OSM, oncostatin M

First, we used a large number of individual donors to reproduce the effect of C5a on OSM, as there is often a high inter‐individual variability (Figure 1b). After this initial experiment, we defined a sample size of no more than 12 individual donors tested in each subsequent experiment, based on our experience with the in vitro experiments, taking the inter‐individual variability into account. Based on the cell viability and cell morphology, we had to exclude some experiments with macrophages from few donors. Therefore, in some figures, we show less than 12 experiments, but in every case, the sample size is larger as 5.

PBMCs were separated by density gradient centrifugation on lymphoprep (Fresenius Kabi Norge AS, Oslo, Norway). With a seeding density of 1 × 10^6^ cells per well, PBMCs were plated in a 24‐well plate in Iscoves Medium supplemented with AB serum (2.5% v/v). To attach the monocytes, cells were incubated for 2 hr at 5% CO_2_ and 37°C. Non‐adherent cells were removed by vigorously washing of adherent cells three times with PBS. An appropriate amount of RPMI 1640, supplemented with 2‐mM l ‐glutamine, 100 mg·ml^−1^ penicillin/streptomycin, 12 mM HEPES, and 5 % v/v human AB serum (Sigma Aldrich; all other media components from Biochrom, Berlin, Germany), and 10 ng·ml^−1^ granulocyte macrophage colony‐stimulating factor (GM‐CSF; R&D, San Diego, CA, USA) was added.

### 2.2 Cell culture, differentiation of M1 and M2 macrophages

Cells were differentiated to M1 and M2 macrophages under the following experimental settings:

Cells were cultured in the presence of GM‐CSF during the differentiation process to M1 macrophages. M2 macrophages were differentiated from primary human monocytes in the presence of macrophage colony‐stimulating factor (M‐CSF) (10 ng·ml^−1^). M2 macrophages were activated by IL‐4 (20 ng·ml^−1^) for 24 hr. At Day 5, another 50% by volume of fresh medium containing GM‐CSF or M‐CSF, respectively, was added. At Day 8, the medium was completely changed. At Day 10, the differentiation process was controlled: M1 macrophages appeared as adherent cells showing a typical morphology with a prominent nucleus, spread out cytoplasm, and a couple of pseudopodia. Fully differentiated M1 macrophages were positive for the intracellularly expressed macrophage differentiation marker CD68. They did not express the scavenger receptor CD163 that is specific for M2 macrophages and was expressed on the M2 macrophages cultured with M‐CSF. When the cells were activated with IFN‐γ (200 ng·ml^−1^) and LPS (50 ng·ml^−1^), we observed a moderate down‐regulation of CD68 and a high up‐regulation of CD80 reflecting the commonly accepted marker profile for activated M1 macrophages (Biswas & Mantovani, 2010). The expression of CD163 was not induced in response to the activating cytokines (Figure 3a–d).

### 2.3 Stimulation of fully differentiated M1 macrophages

**Figure 2:**
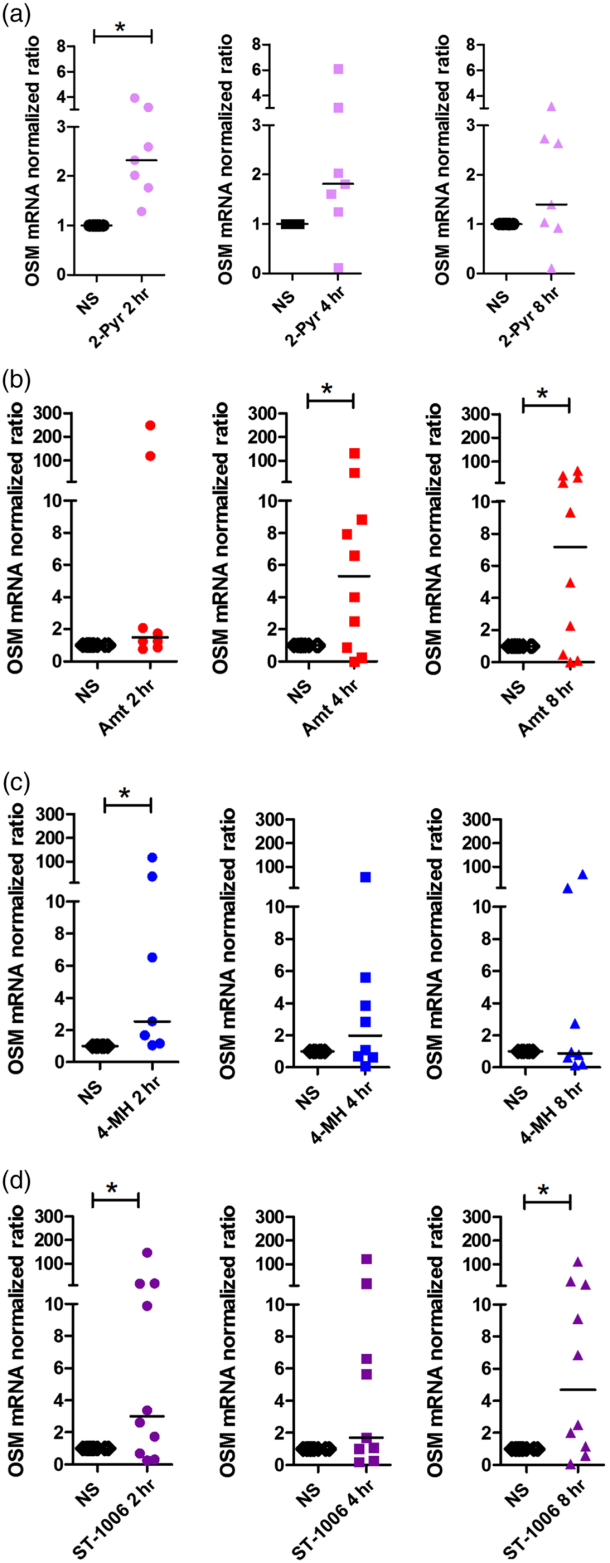
The specific histamine receptor agonists targeting H_1_, H_2_ and H_4_ receptors up‐regulate OSM mRNA expression with different time‐courses in monocyte derived human M1 macrophages. Primary human monocytes were obtained from peripheral blood mononuclear cells after 2‐hr adherence. M1 macrophages were differentiated from primary human monocytes in the presence of GM‐CSF (10 ng·ml^−1^) for 10 days and then stimulated with 2‐pyridylethylamine (H_1_ receptor agonist), amthamine (H_2_ receptor agonist), 4‐methylhistamine (4‐MH; H_2_/H_4_ receptor agonist), and ST‐1006 (H_4_ receptor agonist) for different time periods as indicated. (a) OSM mRNA expression after treatment for different time periods as indicated with 2‐pyridylethylamine (2‐Pyr; *n* = 7 independent donors and experiments). (b) OSM mRNA expression after treatment for different time periods as indicated with amthamine (Amt; *n* = 8 [2 hr] and *n* = 10 [4 and 8 hr] independent donors and experiments). (c) OSM mRNA expression after treatment for different time periods as indicated with 4‐methylhistamine (4‐MH; *n* = 7 [2 hr] and *n* = 8 [4 and 8 hr] independent donors and experiments). (d) OSM mRNA expression after treatment for different time periods as indicated with ST‐1006 (*n* = 10 [2 and 8 hr] and *n* = 9 [4 hr] independent donors and experiments). The amount of the target mRNA relative to the amount of the reference gene, rps 20 mRNA in each stimulated sample was normalized to the amount of the target mRNA relative to the amount of the reference gene in the non‐stimulated sample (calibrator) and expressed as normalized ratio. This was calculated using the comparative Ct method also known as the ΔΔCt method provided by the Software LC 480 (Roche Molecular Biochemicals). Data shown are individual values with medians. *P < .05; significantly different as indicated; Wilcoxon matched‐pairs signed rank test. Ct method, crossing point method; NS, non‐stimulated; OSM, oncostatin M

**Figure 3:**
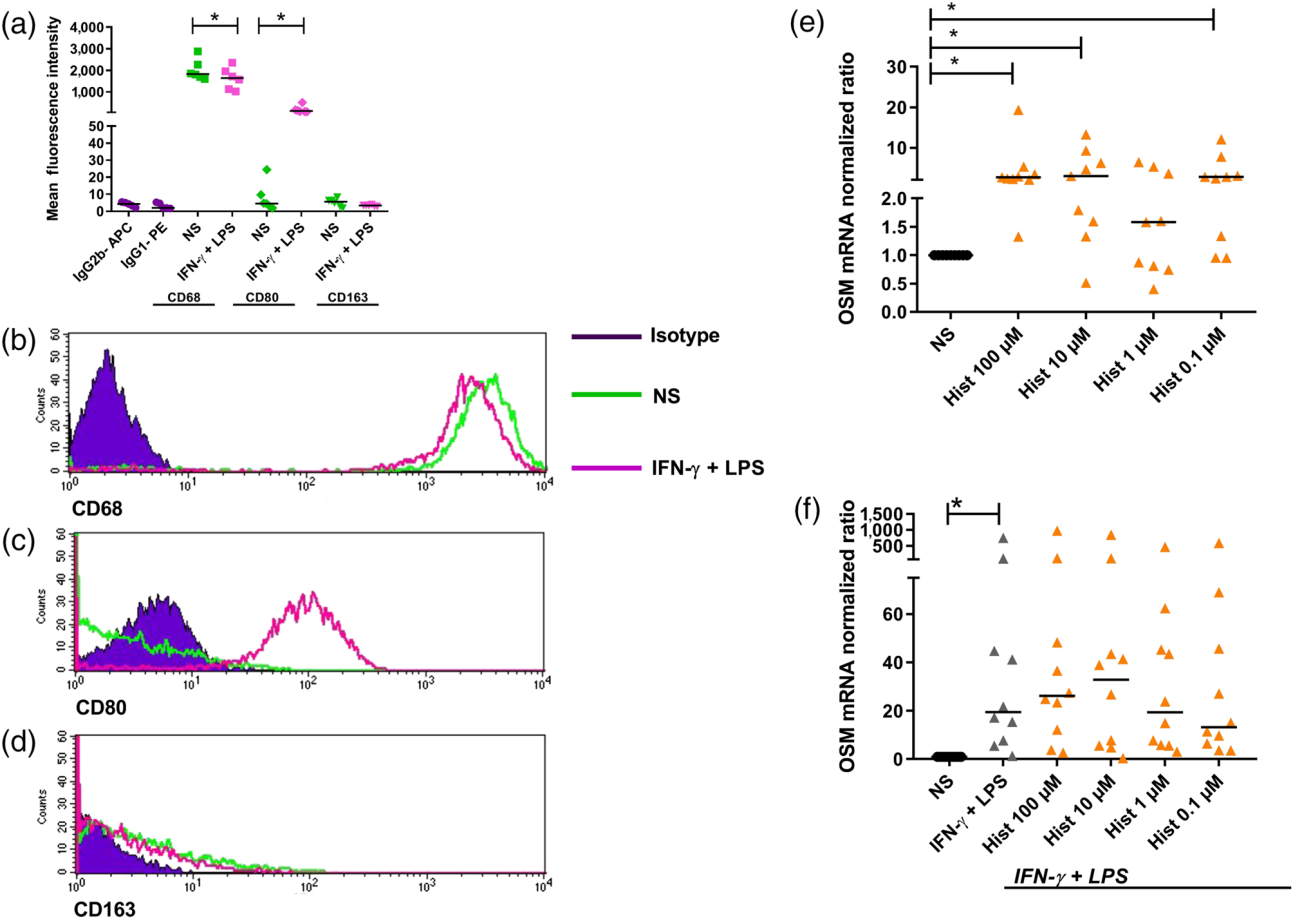
M1 macrophages activated with IFN‐γ + LPS show the commonly accepted marker profile for these cells. Histamine up‐regulates OSM mRNA expression in a dose‐dependent manner in fully differentiated M1 macrophages. Primary human monocytes were obtained from peripheral blood mononuclear cells after 2‐hr adherence. M1 macrophages were differentiated from primary human monocytes in the presence of GM‐CSF (10 ng·ml^−1^) for 10 days. One part was activated with IFN‐γ (200 ng·ml^−1^) and LPS (50 ng·ml^−1^) for additional 24 hr. (a) The M1 marker profile expression of CD68 and CD80 and expression of the M2 marker CD163 were determined by flow cytometry in fully differentiated non‐stimulated and IFN‐γ + LPS‐activated cells to prove that the cells are M1 and not M2 polarized (*n* = 6 independent donors and experiments). Mean fluorescence intensities were calculated. One representative histogram out of six for (b) CD68, (c) CD80, and (d) CD163 is shown for fully differentiated non‐stimulated M1 macrophages, IFN‐γ + LPS‐activated M1 macrophages and the isotype control. (e) OSM mRNA expression in fully differentiated M1 macrophages after treatment with different concentrations of histamine as indicated for 2 hr is depicted (*n* = 9 independent donors and experiments). (f) OSM mRNA expression in fully differentiated M1 macrophages after treatment with different concentrations of histamine as indicated for 2 hr and activated with IFN‐γ (200 ng·ml^−1^) and LPS (50 ng·ml^−1^) for additional 24 hr is depicted (*n* = 10 independent donors and experiments). The amount of the target mRNA relative to the amount of the reference gene, rps 20 mRNA in each stimulated sample was normalized to the amount of the target mRNA relative to the amount of the reference gene in the non‐stimulated sample (calibrator) and expressed as normalized ratio. This was calculated using the comparative Ct method also known as the ΔΔCt method provided by the Software LC 480 (Roche Molecular Biochemicals). Data shown are individual values with medians. In ( e) *P < .05; significantly different as indicated; Friedman Dunn's Multiple Comparisons test selected pairs. In (a) and (f), *P < .05; significantly different as indicated; Wilcoxon matched‐pairs signed rank test. Hist, histamine; NS, non‐stimulated; OSM, oncostatin M

Fully differentiated M1 macrophages were stimulated with C5a (10 ng·ml^−1^), histamine, or specific histamine receptor agonists targeting the H_1_, H_2_ or H_4_ receptor (10 μM), as listed below (Materials) for different time periods 2, 4, and 8 hr as indicated in Figures 1 and 2. For concentration–response experiments, M1 macrophages were stimulated with histamine over a concentration range (100 ‐ 0.1 μM) for 2 hr, and cells were lysed by RNA lysis buffer (Figure 3e), or for 24 hr and then re‐stimulated in the same concentrations, supernatants were taken after 48 hr for OSM elisa (Figure 5a). In the other model, the cells were stimulated with the agonists as indicated over the same concentration range and after 2 hr (Figure 3f and 5b) or after 24 hr (Figure 5c), the cells were activated with IFN‐γ (200 ng·ml^−1^) + LPS (50 ng·ml^−1^) for further 24 hr.

Our stimulation protocol represents an objective, non subjective assay and blinding is not applicable to these types of in vitro studies. The expression of OSM was analysed at mRNA and protein level.

### 2.4 Stimulation of normal human epidermal keratinocytes (NHEKs) with supernatants from histamine‐stimulated M1 macrophages

The use of normal human epidermal keratinocytes (NHEKs) generated from human foreskin or outer root sheath keratinocytes (ORSKs) in research studies investigating inflammatory skin diseases was approved by the local ethics committee of the Hannover Medical School (Vote 2603‐2015) and was conducted according to the declaration of Helsinki Principles.

The investigation of the role of the histamine receptors in inflammatory diseases was approved by the local ethics committee of the Hannover Medical School (Vote 4253) and was conducted according to the declaration of Helsinki Principles.

NHEKs were prepared from juvenile foreskin, as described previously (Glatzer et al., 2013; Zeitvogel et al., 2012). Briefly, the foreskin was cut into pieces and incubated overnight at 37°C in 2.4 U of Dispase II (Roche, Mannheim, Germany). The next day, the epidermis was separated from the dermis and placed for 20 min at 37°C in EDTA (0.02%)–trypsin (0.05%) solution (PAN‐Biotech, Aidenbach, Germany). After stopping the trypsin reaction by addition of FCS (PromoCell, Heidelberg, Germany), the cell suspension was filtered through a sterile gauze (40 mm) and washed twice with PBS. The obtained single‐cell suspension of NHEKs was incubated in the serum‐free growth medium Keratinocyte Growth Medium 2 Kit (PromoCell) at 37°C in a humidified atmosphere containing 5% CO2. Normally, when cells in passages 3 to 7 reached 70–80% confluence, they were used for experiments or further passaged. We periodically check the typical morphology (see the photograph in Figure 7) and the expression of keratinocyte specific marker proteins at mRNA level (Zeitvogel et al., 2012).

NHEKs were seeded in 48‐well plates at a density of 1.5 × 10^4^ cells per well in 100‐μl Keratinocyte Growth Medium and treated with 100 μl of histamine‐stimulated M1 macrophage supernatants as indicated. To show that NHEKs react with STAT3 phosphorylation upon OSM stimulation, we stimulated the NHEKs directly with rh OSM (0.1 ng·ml^−1^). To analyse the phosphorylation of STAT3, NHEKs were incubated for 20 min with respective M1 macrophage supernatants following phospho‐STAT3 staining using the intracellular fixation/methanol protocol with monoclonal mouse IgG2b antibody against phospho‐STAT3 (Tyr^705^; 1 μg·ml^−1^; Thermo Fischer Scientific, Waltham, Massachusetts, USA) and respective mouse isotype (IgG2b, 1 μg·ml^−1^) control in the same concentration.

### 2.5 Flow cytometric analysis of M1 marker CD68, CD80, and M2 marker CD163

The antibody‐based procedures used in this study comply with the recommendations made by the *British Journal of Pharmacology*. Macrophages were carefully scraped from the culture plates and seeded (5 × 10^5^ cells per well) into 96‐well plates. Fc receptors were blocked by incubation in a buffer containing 10 μg·ml^−1^ heat‐aggregated human IgG (Sigma, Deisenhofen, Germany). An extracellular epitope of CD163 was stained with anti‐human CD163‐PE (mouse monoclonal IgG1, 1 μg/100 μl; BioLegend, San Diego, CA, USA; RRID:AB_893269); the respective isotype control IgG1 from BioLegend was tested in parallel in the same concentration. The marker CD80 was stained with anti‐human CD80‐PE (mouse monoclonal IgG1, 1 μg/100 μl; BioLegend; RRID:AB_314504); the respective isotype control IgG1 from BioLegend was tested in parallel in the same concentration. Then the cells were fixed, permeabilized using the BD Cytofix/Cytoperm fixation/permeabilization kit (BD Bioscience) and CD68, which is intracellularly expressed in cytoplasmic granules, and was stained with anti‐human CD68‐APC (mouse monoclonal IgG2b; 1 μg·ml^−1^; BioLegend; RRID:AB_10567107); the respective isotype control (IgG2b, 1 μg·ml^−1^; BioLegend) was tested in parallel. Sample acquisition was performed by flow cytometry (FACS Calibur, Becton Dickinson, Heidelberg, Germany), and mean fluorescence intensities were calculated by CellQuest Pro software (Becton Dickinson). To assess cell viability, macrophages were incubated with Annexin V‐FITC and propidium iodide for 15 min (BD Bioscience) and analysed by flow cytometry (FACS Calibur, Becton Dickinson).

### 2.6 Flow cytometric analysis of intracellular expression of phospho‐STAT3

After treatment with supernatants or rh OSM (Immunotools) as positive control for 20 min, keratinocytes were detached by incubating with 0.0025% EDTA (PAN Biotech, Aidenbach, Germany) for 3–5 min following an incubation period with HyQTase (Perbio, Bonn, Germany) up to 10 min. For intracellular staining, keratinocytes were fixed and permeabilized using Cytofix/Cytosperm (BD Bioscience, Heidelberg, Germany) according to the manufacturer's instructions. For staining of intracellular phosphorylated signalling proteins such as STAT3, the fixation is followed by treatment of cells with ice‐cold 90–100% methanol for at least 30 min at 2–8°C. APC‐labelled phospho‐STAT3 monoclonal mouse IgG2b antibody (Thermo Fischer Scientific, Waltham, Massachusetts; 1 μg·ml^−1^) was used for intracellular staining of phosphorylated STAT3. Control staining was performed with the matched isotype used in the same final concentration as the staining antibody (APC‐labelled mouse IgG2b (BioLegend, San Diego, USA). The matched isotype showed no unspecific binding.

Sample acquisition was performed by flow cytometry (FACS Calibur, Becton Dickinson, Heidelberg, Germany), and mean fluorescence of gated cells was calculated by CellQuest Pro software (Becton Dickinson).

### 2.7 Real‐time quantitative PCR

Total RNA was isolated using the RNeasy kit (Qiagen, Hilden, Germany) according to the manufacturer's instructions. The cDNA was synthesized by reverse transcription (QuantiTect reverse transcription kit, Qiagen, Germany). Real‐time quantitative PCR was performed according to the MIQE guidelines with Quantitect® primer assays for OSM (QT00209286), and ribosomal protein S20 (rps 20; QT00079247) using SYBR® Green according to the manufacturer's instructions (Qiagen, Hilden, Germany) using the LightCycler 480.

The amount of the target mRNA relative to the amount of the reference gene, rps 20 mRNA, in each stimulated sample was normalized to the amount of the target mRNA relative to the amount of the reference gene in the non‐stimulated sample (calibrator) and expressed as normalized ratio. This was calculated using the comparative Ct method also known as the ΔΔCt method provided by the Software LC 480 (Roche Molecular Biochemicals).

The regulation of the mRNA expression was given as normalized ratio to show the variability of individual gene regulation of every donor.

### 2.8 Data and statistical analysis

The data and statistical analysis comply with the recommendations of the *British Journal of Pharmacology* on experimental design and analysis in pharmacology (Curtis et al., 2018). For statistical analyses, the software GraphPad Prism Version 8.0 was used (GraphPad software, San Diego, CA, USA, RRID:SCR_002798). First, we performed methods to test the normal Gaussian distribution of the data. In all our experiments due to the individual variations of the data, the normality tests failed. The non‐parametric tests Wilcoxon matched‐pairs signed rank test or Friedman Dunn's Multiple Comparisons test selected pairs were used and the medians are shown in the graphs. A *P* value < .05 was regarded as statistically significant.

### 2.9 Materials

The following histamine receptor ligands and stimuli were used in this study: histamine (ALK‐Abello, Madrid, Spain) as agonist for all histamine receptors; 2‐pyridylethylamine (Tocris Bioscience, Bristol, UK) as selective H1 receptor agonist; amthamine (Tocris Bioscience, Bristol, UK) as selective H2 receptor agonist; 4‐methylhistamine as a H2 /H4 receptor agonist; ST‐1006 (Institute of Pharmaceutical and Medicinal Chemistry, Heinrich Heine University, Duesseldorf, Germany) as H4 receptor agonist (Sander et al., 2009); clemastine (Tocris) as selective H1 receptor antagonist; ranitidine (Tocris) as selective H2 receptor antagonist; and JNJ7777120 (Sigma Aldrich, Deisenhofen, Germany) as selective H4 receptor antagonist. All histamine receptor ligands were used at a concentration of 10 μM. In concentration–response experiments, the histamine receptor ligands were used in the range of 0.1 ‐ 100 μM. In extensive previous studies, we have shown that 10 μM is the optimal concentration to demonstrate and reproduce robust H4 receptor agonist mediated effects (Gschwandtner, Koether, Werfel, Stark, & Gutzmer, 2013). C5a (10 ng·ml−1; Sigma, Deisenhofen, Germany); recombinant human rh OSM (1 ng·ml−1) ImmunoTools, Friesoythe, Germany); GM‐CSF (10 ng·ml−1), IFN‐γ (200 ng·ml−1) (R&D, San Diego, CA, USA); LPS (50 ng·ml−1) Sigma Aldrich.

### 2.10 Nomenclature of targets and ligands

Key protein targets and ligands in this article are hyperlinked to corresponding entries in http://www.guidetopharmacology.org, the common portal for data from the IUPHAR/BPS Guide to PHARMACOLOGY (Harding et al., 2018), and are permanently archived in the Concise Guide to PHARMACOLOGY 2017/18 (Alexander, Christopoulos et al., 2017; Alexander, Fabbro et al., 2017; Alexander, Kelly et al., 2017).

## 3 RESULTS

### 3.1 Both histamine and C5a up‐regulate OSM mRNA expression but with different kinetics in monocyte‐derived human M1 macrophages

To evaluate if histamine has effects similar to those previously shown for C5a on OSM production in human monocyte derived M1 macrophages, we stimulated GM‐CSF‐treated M1 macrophages with C5a (10 ng·ml^−1^) as positive control stimulus or with histamine (10 μM) for 2, 4, and 8 hr as indicated in Figure 1a. Apart from C5a, histamine also up‐regulated OSM mRNA expression. However, C5a stimulation resulted in a significant up‐regulation of OSM mRNA expression after 2 hr whereas histamine showed significant effects after 2‐ and 4‐hr stimulation (Figure 1b,c).

### 3.2 The specific histamine receptor agonists targeting H_1_, H_2_, and H_4_ receptors up‐regulate OSM mRNA expression with different kinetics in monocyte‐derived human M1 macrophages

In vitro differentiation of monocytes in the presence of GM‐CSF led to generation of M1 macrophages, which express the H_1_ , H_2_ and H_4_ receptors at mRNA level (Mommert, Ratz, Stark, Gutzmer, & Werfel, 2018). Therefore, we stimulated the fully differentiated M1 macrophages with specific agonists targeting the H_1_, H_2_ and H_4_ receptors. The H_1_ receptor agonist, 2‐pyridylethylamine moderately (2.3‐fold median) but significantly up‐regulated OSM mRNA expression after a stimulation period of 2 hr. Amthamine (H_2_ receptor agonist) up‐regulated OSM mRNA expression to a higher degree when compared with the other histamine receptor agonists after a stimulation period of 4 (5.3‐fold median) and 8 hr (7.2‐fold median). 4‐Methylhistamine (4‐MH; H_2_/H_4_ receptor agonist) up‐regulated OSM mRNA expression (2.5‐fold median) after a stimulation period of 2 hr. ST‐1006 (H_4_ receptor agonist) also moderately but significantly up‐regulated OSM mRNA expression after 2 (3.0‐fold median) and 8 hr (4.7‐fold median; Figure 2a–d).

### 3.3 Histamine up‐regulates OSM mRNA expression in a dose‐dependent manner in fully differentiated M1 macrophages: IFN‐γ + LPS‐activated M1 macrophages express higher levels of OSM mRNA than those in non‐activated cells

We stimulated fully differentiated M1 macrophages with different concentrations of histamine (0.1 – 100‐μM) for 2 hr. OSM mRNA expression was significantly up‐regulated in samples stimulated with 100, 10, and 0.1 μM histamine (Figure 3e). This fits to the observation of Kastl et al. (2008) only in part. They showed in contrast to the effective concentrations of histamine that C5a up‐regulated expression of OSM mRNA most effectively at 1 μM. In another set of experiments, M1 macrophages were left non‐stimulated or activated with IFN‐γ + LPS for 24 hr. IFN‐γ + LPS‐activated M1 macrophages express higher levels of OSM mRNA when compared non‐activated cells (Figure 3f). There was a non‐significant trend for histamine (10 μM) to potentiate the IFN‐γ + LPS‐induced expression of OSM mRNA. M1 macrophages showed the commonly accepted marker profile for these cells, expressing CD68 in a moderate, lower level but expressing CD80 highly up‐regulated, when compared with non‐activated cells. The M2 marker CD163 was expressed neither in fully differentiated nor in activated M1 macrophages (Figure 3a–d).

### 3.4 The up‐regulation of OSM mRNA expression by histamine and specific agonists is blocked by pre‐incubation with antagonists targeting H_1_, H_2_ and H_4_ receptors in M1 macrophages

**Figure 4:**
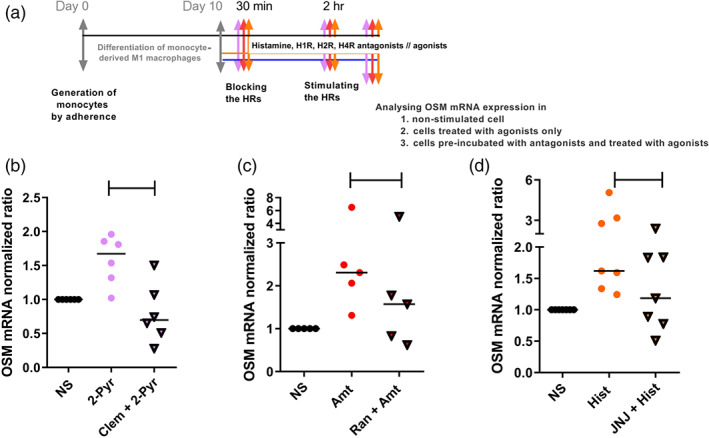
The up‐regulation of OSM mRNA expression by histamine or specific histamine receptor agonists in M1 macrophages is blocked by pre‐incubation with antagonists targeting H_1_, H_2_ and H_4_ receptors. (a) Primary human monocytes were obtained from peripheral blood mononuclear cells after 2‐hr adherence. M1 macrophages were differentiated from primary human monocytes in the presence of GM‐CSF (10 ng·ml^−1^) for 10 days. The cells were pre‐incubated with antagonists targeting the H_1_ receptor (clemastine; Clem), H_2_ receptor (ranitidine; Ran), and H_4_ receptor (JNJ7777120; JNJ) for 30 min before stimulation and then stimulated with 2‐pyridylethylamine (2‐Pyr; H_1_ receptor agonist), amthamine (Amt; H_2_ receptor agonist), and histamine for additional 2 hr. (b) OSM mRNA expression after treatment with H_1_ receptor antagonist and agonist (*n* = 6 independent donors and experiments). (c) OSM mRNA expression after treatment with H_2_ receptor antagonist and agonist (*n* = 5 independent donors and experiments). (d) OSM mRNA expression after treatment with H_4_ receptor antagonist and histamine (Hist; *n* = 7 independent donors and experiments). The amount of the target mRNA relative to the amount of the reference gene, rps 20 mRNA in each stimulated sample was normalized to the amount of the target mRNA relative to the amount of the reference gene in the non‐stimulated sample (calibrator) and expressed as normalized ratio. This was calculated using the comparative Ct method also known as the ΔΔCt method provided by the Software LC 480 (Roche Molecular Biochemicals). Data shown are individual values with medians. None of the antagonists significantly inhibited the response to the agonists (*P* values ranged from .06 to .08; Wilcoxon matched‐pairs signed rank test), although there was a consistent trend towards inhibition. Ct method, crossing point method; Hist, histamine; NS, non‐stimulated; OSM, oncostatin M

To reliably demonstrate that the up‐regulation of OSM mRNA expression in human monocyte‐derived M1 macrophages is mediated via the H_1_, H_2_ and H_4_ receptors, as shown in Figure 2, we pre‐incubated GM‐CSF‐treated M1 macrophages with antagonists targeting the H_1_, H_2_ and H_4_ receptors before stimulation. Up‐regulation of OSM mRNA expression by specific H_1_ and H_2_ receptor agonists was in part inhibited by pre‐incubation with the specific antagonists, clemastine or ranitidine for the H_1_ and H_2_ receptor, respectively (Figure 4b,c). The histamine‐mediated up‐regulation of OSM mRNA was partly inhibited by pre‐incubation with the H_4_ receptor‐specific antagonist JNJ7777120 (Figure 4d). The histamine‐induced up‐regulation of OSM mRNA expression was not consistently blocked by H_1_ or H_2_ receptor antagonists.

### 3.5 Histamine up‐regulates OSM protein production in fully differentiated and in IFN‐γ + LPS‐activated M1 macrophages

**Figure 5:**
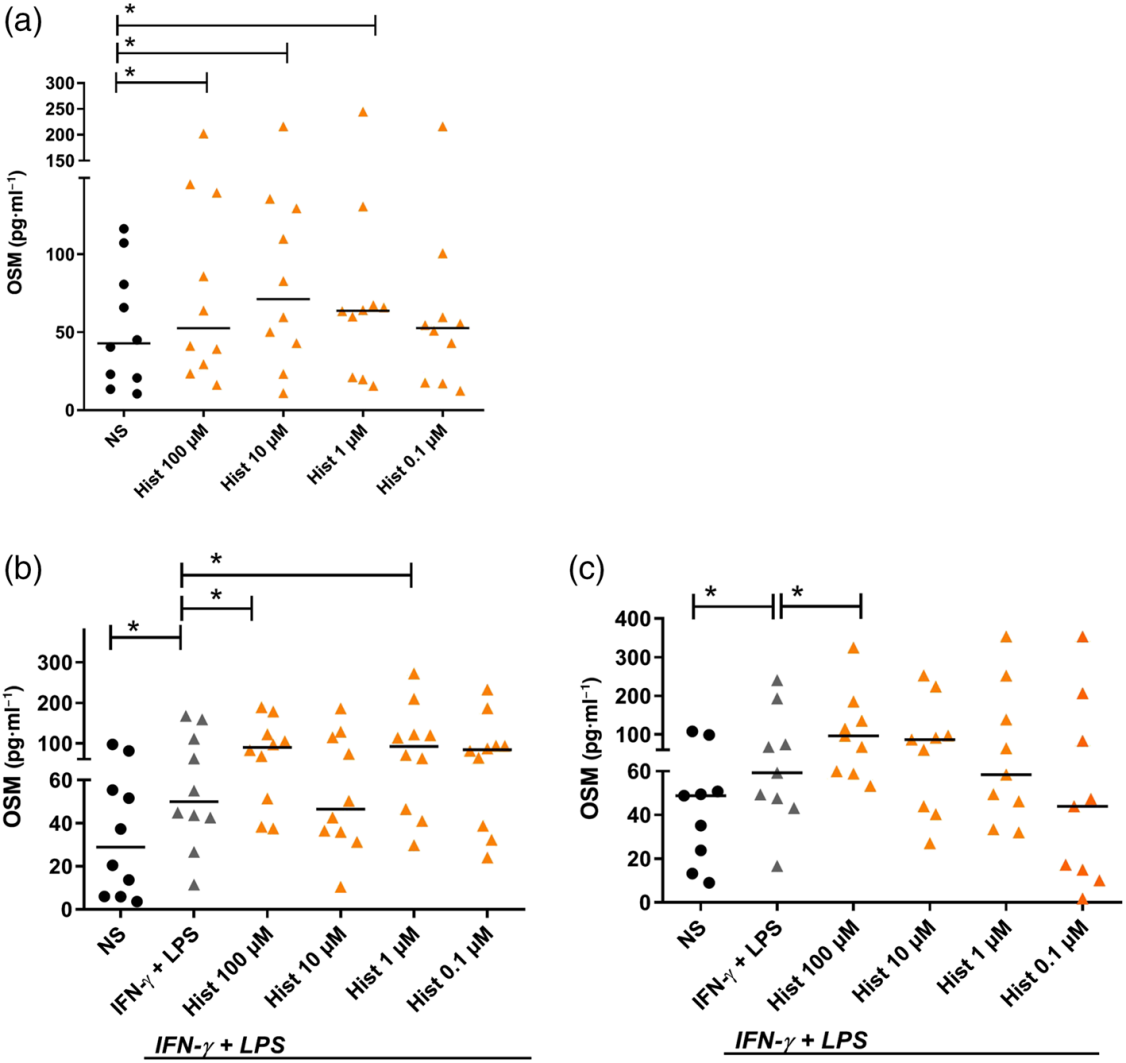
Histamine up‐regulates OSM protein production in non‐activated and in IFN‐γ + LPS‐activated M1 macrophages in different doses. Primary human monocytes were obtained from peripheral blood mononuclear cells after 2‐hr adherence. (a) M1 macrophages were differentiated from primary human monocytes in the presence of GM‐CSF (10 ng·ml^−1^) for 10 days and then stimulated with histamine in different concentrations as indicated for 24 hr and re‐stimulated in the same concentrations for additional 24 hr. M1 macrophages were non‐stimulated or stimulated with histamine in different concentrations as indicated for (b) 2 hr or (c) 24 hr and then activated with IFN‐γ (200 ng·ml^−1^) and LPS (50 ng·ml^−1^) for additional 24 hr. OSM protein production was analysed by elisa. Data shown are individual values with medians from (a, b) *n* = 10 independent donors and experiments; (c) *n* = 9 independent donors and experiments. In (b) and (c),*P < .05; significantly different (NS and IFN‐γ + LPS); Wilcoxon matched‐pairs signed rank test. In (a), (b) and (c), *P <.05, significantly different as indicated; Friedman Dunn's Multiple Comparisons test selected pairs. Hist, histamine; NS, non‐stimulated; OSM, oncostatin M

We stimulated the fully differentiated M1 macrophages with different concentrations of histamine (0.1 – 100‐μM) for 24 hr. The cells were re‐stimulated with respective stimuli. Twenty‐four hours later, supernatants were taken, and OSM protein production was measured by elisa. OSM protein production was significantly up‐regulated in samples stimulated with histamine (100, 10, and 1 μM; Figure 5a). In further experiments, M1 macrophages were left non‐stimulated or stimulated for 2 hr (Figure 5b) or 24 hr (Figure 5c) with histamine (0.1 – 100‐μM) and then activated with IFN‐γ + LPS for further 24 hr. Activation with IFN‐γ + LPS led to a significant up‐regulation of OSM protein production that was potentiated by histamine, at 100 and 1 μM when stimulated for 2 hr before activation by IFN‐γ + LPS; Figure 5b) and at 100 μM when stimulated for 24 hr before activation by IFN‐γ + LPS; Figure 5c).

### 3.6 Histamine up‐regulates IL‐6 protein production in fully differentiated M1 macrophages: IFN‐γ + LPS‐activated M1 macrophages express higher levels of IL‐6, TNF‐α, and IL‐12 than those in non‐stimulated M1 macrophages

**Figure 6:**
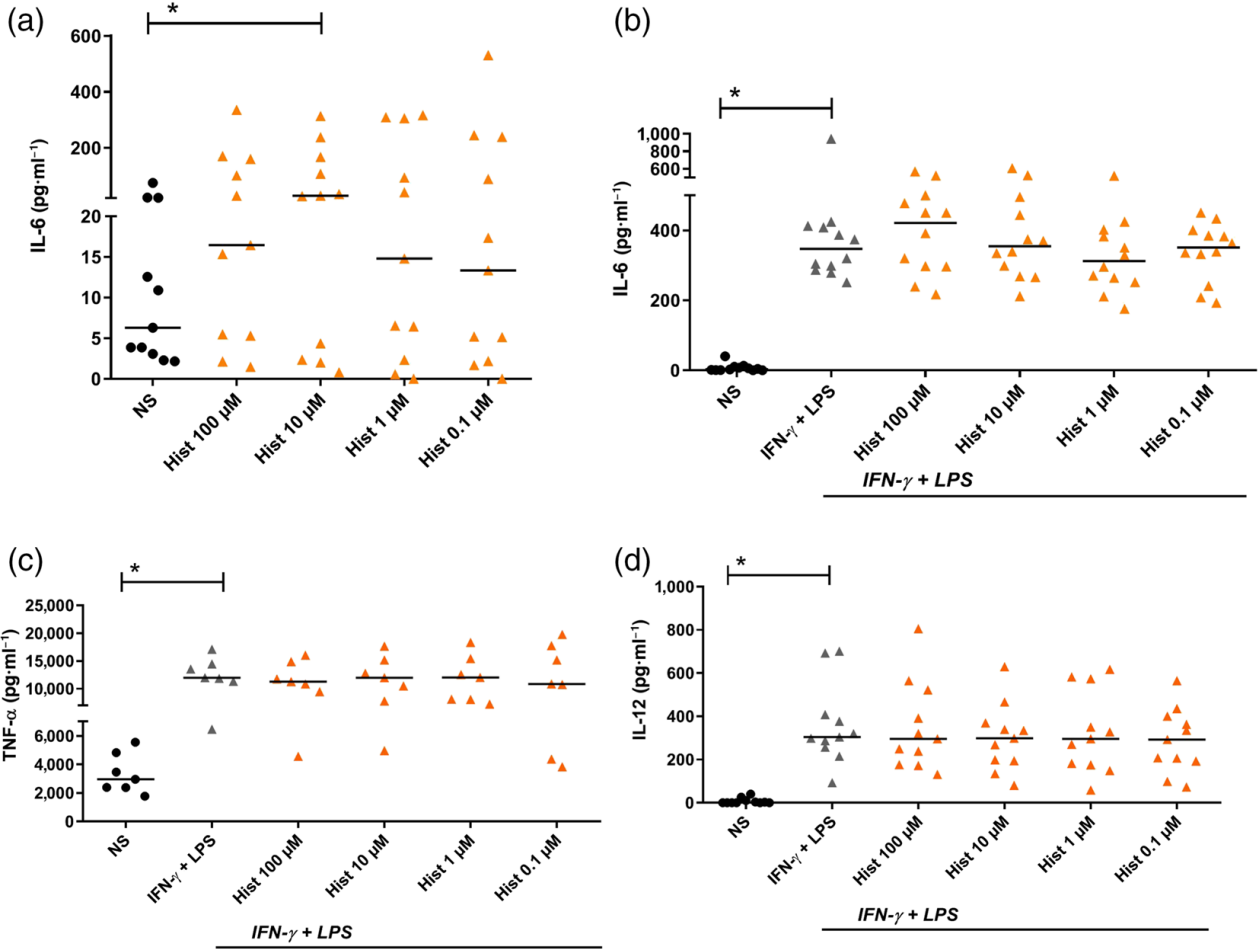
Histamine up‐regulates IL‐6 production in fully differentiated M1 macrophages. IFN‐γ + LPS‐activated M1 macrophages produce higher levels of IL‐6, TNF‐α and IL‐12 protein when compared to non‐activated cells. Histamine did not potentiate IL‐6, TNF‐α and IL‐12 protein production. Primary human monocytes were obtained from peripheral blood mononuclear cells after 2‐hr adherence. M1 macrophages were differentiated from primary human monocytes in the presence of GM‐CSF (10 ng·ml^−1^) for 10 days. (a) M1 macrophages were left non‐stimulated or stimulated with histamine in different concentrations as indicated for 24 hr and re‐stimulated in the same concentrations for additional 24 hr. M1 macrophages were left non‐stimulated or stimulated with histamine in different concentrations as indicated for 24 hr and then activated with IFN‐γ (200 ng·ml^−1^) and LPS (50 ng·ml^−1^) for additional 24 hr. Secretion of (b) IL‐6 protein, (c) TNF‐α, and (d) IL‐12 protein was measured by elisa technique. Data shown are individual values with medians from (a) *n* = 11 independent donors and experiments; (b) *n* = 12 independent donors and experiments; (c) *n* = 7 independent donors and experiments; (d) *n* = 11 independent donors and experiments. In (a), *P < .05; significant differences between non‐stimulated and histamine stimulated cells; Friedman Dunn's Multiple Comparisons test selected pairs. In (b), (c) and (d), *P < .05, significant differences as indicated; Wilcoxon matched‐pairs signed rank test. Hist, histamine; NS, non‐stimulated; OSM, oncostatin M

We stimulated fully differentiated M1 macrophages with different concentrations of histamine (0.1 – 100‐μM) for 24 hr. The cells were re‐stimulated with respective stimuli. Twenty‐four hours later, supernatants were taken. IL‐6, TNF‐α, and IL‐12 protein production were measured by elisa. IL‐6 protein production was significantly up‐regulated in samples stimulated with histamine (10 μM ;Figure 6a). IL‐12 protein was barely detected in the unstimulated supernatants (Figure 6d). In another set of experiments, M1 macrophages were left non‐stimulated or stimulated for 24 hr with histamine (0.1 – 100‐μM) and then activated with IFN‐γ + LPS for further 24 hr. Activation with IFN‐γ + LPS led to a significant up‐regulation of IL‐6, TNF‐α, and IL‐12 protein production, which was not further regulated by histamine (Figure 6b–d).

As comparison, we differentiated monocyte‐derived M2 macrophages in the presence of M‐CSF (10 ng·ml^−1^). We stimulated the M2 macrophages with IL‐4 (20 ng·ml^−1^) to polarize the cells into M2a macrophages. After 24 hr, the cells were stimulated with different histamine receptor agonists targeting the H_1_, H_2,_ and H_4_ receptors . OSM production in this subset of macrophages was not regulated either by IL‐4 or by the histamine receptor agonists (Figure S1).

### 3.7 Normal human epidermal keratinocytes (NHEKs) incubated with supernatants from histamine‐treated M1 macrophages show an increased STAT3 phosphorylation

**Figure 7:**
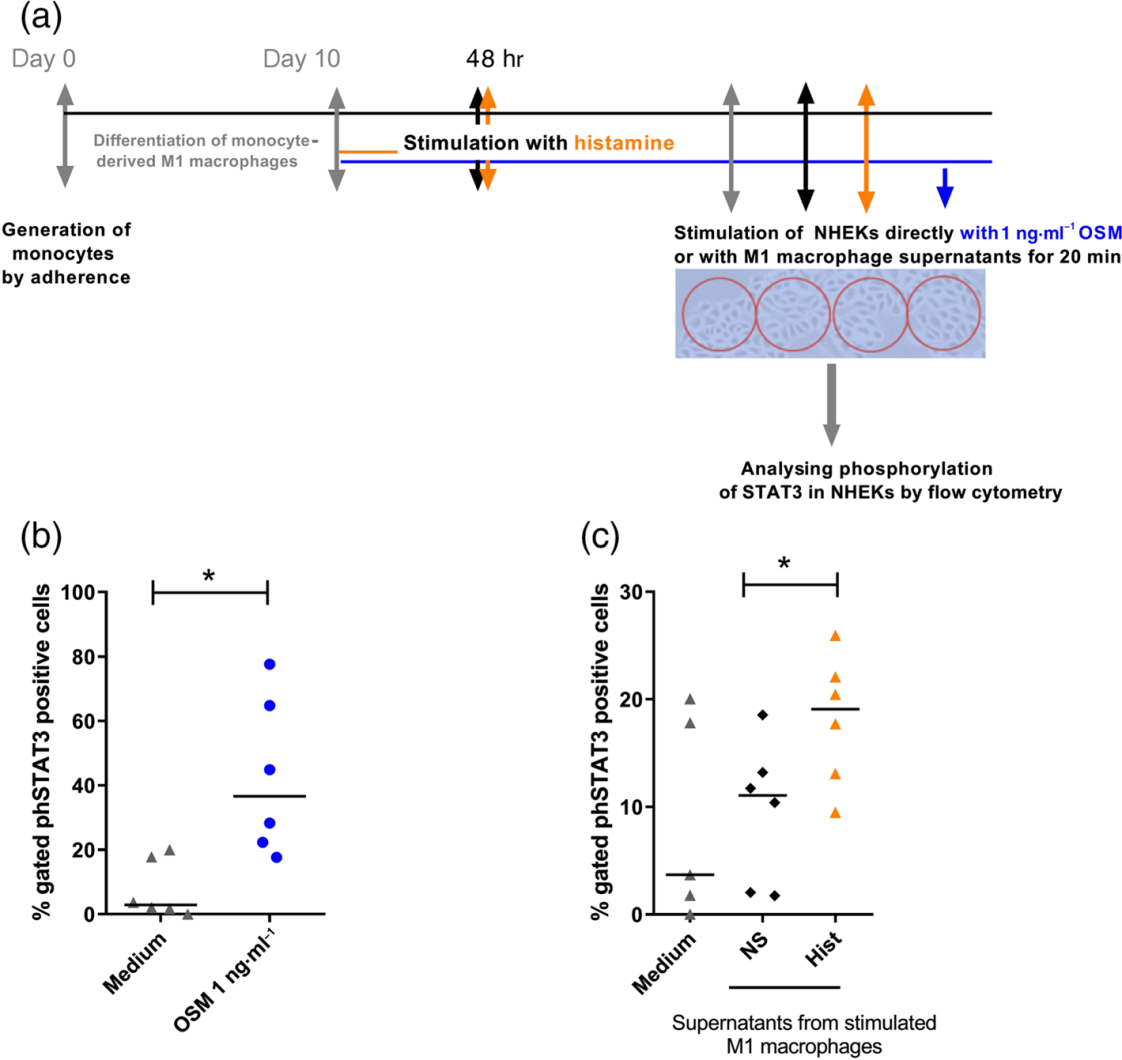
Normal human epidermal keratinocytes (NHEKs) incubated with supernatants from histamine‐treated M1 macrophages show increased STAT3 phosphorylation. Primary human monocytes were obtained from peripheral blood mononuclear cells after 2‐hr adherence. M1 macrophages were differentiated from primary human monocytes in the presence of GM‐CSF (10 ng·ml^−1^) for 10 days and stimulated with histamine (10 μM) for 24 hr and re‐stimulated for additional 24 hr. The supernatants of stimulated M1 macrophages were added to the cultures of NHEKs for 20 min. (b) Phosphorylation of STAT3 was analysed in rh OSM (1 ng·ml^−1^) stimulated NHEKs (direct stimulation of NHEKs) by flow cytometry. (c) Phosphorylation of STAT3 was analysed in NHEKs stimulated with supernatants from M1 macrophages, as indicated, by flow cytometry. Data shown are individual values with medians from *n* = 6 independent donors and experiments. *P < .05; significantly different as indicated; Wilcoxon matched‐pairs signed rank test. Hist, histamine; NS, non‐stimulated; OSM, oncostatin M, NHEKs, normal human epidermal keratinocytes

NHEKs were incubated with rh OSM (1 ng·ml^−1^) as positive control and with supernatants from histamine‐stimulated M1 macrophages for 20 min, followed by intracellular phospho‐STAT3 staining with an APC‐labelled phospho‐STAT3 monoclonal mouse IgG2b antibody (Figure 7a). Flow cytometric analysis revealed that the direct stimulation with OSM activated STAT3 phosphorylation in NHEKs (Figure 7b). Supernatants from M1 macrophages that were stimulated with histamine showed a significantly higher induction of STAT3 phosphorylation when compared to supernatants from non‐stimulated M1 macrophages (Figure 7c).

## 4 DISCUSSION

Human monocyte‐derived M1 macrophages originate in a micro‐environment containing GM‐CSF, IFN‐γ, or bacterial products such as LPS. These so‐called classically activated M1 macrophages act as one of the first lines of resistance against pathogens by releasing large amounts of pro‐inflammatory cytokines and chemokines, thereby driving the polarization and recruitment of Th1 cells and amplifying a type 1 response (Biswas & Mantovani, 2010; Guihard et al., 2015; Murray et al., 2014; Murray & Wynn, 2011). Among the cytokines produced by M1 macrophages in an inflammatory context, OSM is also up‐regulated in response to LPS, GM‐CSF, or PGE_2_ (Repovic & Benveniste, 2002; Suda et al., 2002). OSM is a potent activator of cytokine, chemokine, or antimicrobial peptide production, as well as inducing migration in NHEKs (Finelt et al., 2005). This may play a role in the course of inflammatory skin diseases where the OSM type II receptor is expressed on keratinocytes (Boniface et al., 2007).

On one hand, an earlier study demonstrated that the pro‐inflammatory mediator OSM was up‐regulated by the anaphylatoxin C5a in human monocyte‐derived M1 macrophages (Kastl et al., 2008). On the other hand, C5a shares the characteristics of histamine regarding its properties to modulate an overwhelming immune response by down‐regulating the production of cytokines of the IL‐12 family in a range of inflammatory conditions (Gutzmer et al., 2005; Okazaki, Hazeki, Izumi, Nigorikawa, & Hazeki, 2011; Wittmann et al., 1999). Furthermore, activation of the transcription factor AP‐1 is induced by both C5a and histamine in macrophages and dendritic cells (Gutzmer et al., 2005; Kastl et al., 2008). Therefore, we suggested that histamine may have immune modulatory functions comparable to C5a in cells of the monocyte lineage. Following our hypothesis, we investigated if histamine exerted effects on OSM production, similar to those shown for C5a in monocyte derived M1 macrophages (Kastl et al., 2008).

We stimulated GM‐CSF differentiated M1 macrophages in parallel with C5a and histamine for different time periods. Analysing mRNA expression, we observed that actually, histamine mirrors the enhanced OSM mRNA expression of C5a after 2‐ and 4‐hr treatment. In our hands, expression of OSM mRNA by C5a was up‐regulated only after a short stimulation time of 2 hr , whereas Kastl et al. showed a significant up‐regulation of OSM mRNA expressions after 2 and 4 hr and with a peak at 8 hr (Kastl et al., 2008). Histamine up‐regulated expression of OSM mRNA over a range of concentrations (0.1 – 100‐μM). As we demonstrated that the mRNA expression levels for H_1_, H_2_ and H_4_ receptors were up‐regulated during the differentiation process of monocyte‐derived human M1 macrophages in the presence of GM‐CSF (Mommert et al., 2018), we treated these cells with agonists and antagonists targeting these three histamine receptors. We observed that the OSM up‐regulation was mainly attributable to H_2_ receptors with moderate contributions from H_1_ and H_4_ receptors.

Furthermore, we could show an up‐regulation of OSM and IL‐6 at the protein level after stimulation with different concentrations of histamine. IFN‐γ + LPS‐activated M1 macrophages secreted higher amounts of OSM, IL‐6, TNF‐α, and IL‐12 when compared to non‐stimulated cells. However, histamine potentiated the IFN‐γ + LPS‐induced OSM production only.

Notably, histamine regulated OSM expression in concentrations ranging from 100 to 0.1 μM. We suggest that this wide concentration range of effective concentrations is due to the different affinities that have been described for the H_1_, H_2_ and H_4_ receptors towards histamine (Panula et al., 2015; Thurmond, 2010). We observed that the up‐regulation of OSM production in response to histamine showed high donor‐dependent variability. In contrast to cell lines, human primary cells reflect more an individual phenotype related to demographic or anamnestic characteristics of the donor. The response of M1 macrophages to histamine also strongly depends on the expression levels of the H_1_, H_2_ and H_4_ receptors, which were differentially and donor dependently regulated on these cells (Mommert et al., 2018).

The presence of both the OSM receptor II and OSM was detected in lesions of cutaneous inflammatory skin diseases. Further, in these studies OSM induced STAT3 phosphorylation and translocation to the nucleus, which in turn is responsible for the OSM‐mediated gene expressions followed by keratinocyte alterations (Boniface et al., 2007; Finelt et al., 2005). We stimulated NHEKs with supernatants from non‐stimulated M1 macrophages and from cells that were treated with histamine. Intracellular staining of phospho‐STAT3 revealed that supernatants from histamine‐treated M1 macrophages have a significantly higher capacity to phosphorylate STAT3 in NHEKs, when compared with non‐stimulated cells. These results may provide evidence that histamine triggers M1 macrophages to produce mediators, which in turn have functional relevance to modulate the activation of keratinocytes. Our study suggests that this effect is mainly attributed to the enhanced secretion of OSM and IL‐6 by histamine.

In human skin, C5a is present in inflammatory conditions and has the ability to trigger the release of histamine from mast cells and basophils (el‐Lati, Dahinden, & Church, 1994). In the present study, we showed that C5a and histamine, in parallel, induced OSM expression in a direct manner, in human M1 macrophages. Our data provide additional hints, in the context of results from previous studies, that C5a and histamine are tracking similar pathways in cells of the monocyte lineage.

Interestingly, the up‐regulation of the pro‐inflammatory mediator OSM or IL‐6 by histamine is in contrast to previous observations where histamine played a more anti‐inflammatory role by reducing the TLR‐induced expression of cytokines of the IL‐12 family (Gschwandtner et al., 2011; Gutzmer et al., 2005). The up‐regulation of OSM production by both mediators may play a critical role in the regulation of pathological immune responses in skin and raises the possibility of novel target strategies in human inflammatory skin diseases. Systemic treatment of inflammatory skin diseases by cyclosporine or corticoids should only be used for short term interventions. Therefore, antagonists targeting the histamine receptors, in particular the histamine H_4_ receptor, which is also involved in OSM up‐regulation will attract increasing attention (Werfel et al., 2018).

## AUTHOR CONTRIBUTIONS

S.M. is the primary author conducting data collection, study design and analysis, interpretation of the data, and writing the first draft of the manuscript. M.H. performed most of the experiments, generating and stimulating the macrophages performing elisa and quantitative PCR. K.S.G. and R.G. made significant contributions to the conception and design of the study. T.W. made substantial contributions to the conception, design of the study, and interpretation of the data. All authors reviewed, revised, and approved the manuscript for publication.

## CONFLICT OF INTEREST

The authors declare no conflicts of interest.

## DECLARATION OF TRANSPARENCY AND SCIENTIFIC RIGOUR

This Declaration acknowledges that this paper adheres to the principles for transparent reporting and scientific rigour of preclinical research as stated in the BJP guidelines for Design & Analysis, and Immunoblotting and Immunochemistry, and as recommended by funding agencies, publishers and other organisations engaged with supporting research.

## Supporting information

Figure S1: OSM production was regulated neither by IL‐4 nor by the histamine receptor agonists in activated M2a macrophages. Primary human monocytes were obtained from PBMCs after 2‐hr adherence. M2 macrophages were differentiated from primary human monocytes in the presence of M‐CSF (10 ng/ml) for 10 days. M2 macrophages were activated by IL‐4 (20 ng/ml) for 24 hr and then stimulated with 2‐pyridylethylamine (H1R agonist), amthamine (H2R agonist), 4‐methylhistamine (4‐MH) (H2R/H4R agonist) and ST‐1006 (H4R agonist) for additional 24 hr. All histamine receptor agonists were used in the concentration of (10 μM). Secretion of OSM protein was measured by elisa technique. (*n* = 12 independent donors and experiments). NS, non stimulated, Hist, histamine, OSM, oncostatin M
