# Supplementary figures and images for: Histamine up‐regulates oncostatin M expression in human M1 macrophages
Source: Br J Pharmacol. 2019 Dec 11;177(3):600–13. doi: 10.1111/bph.14796 (PMC7012943; doi:10.1111/bph.14796)

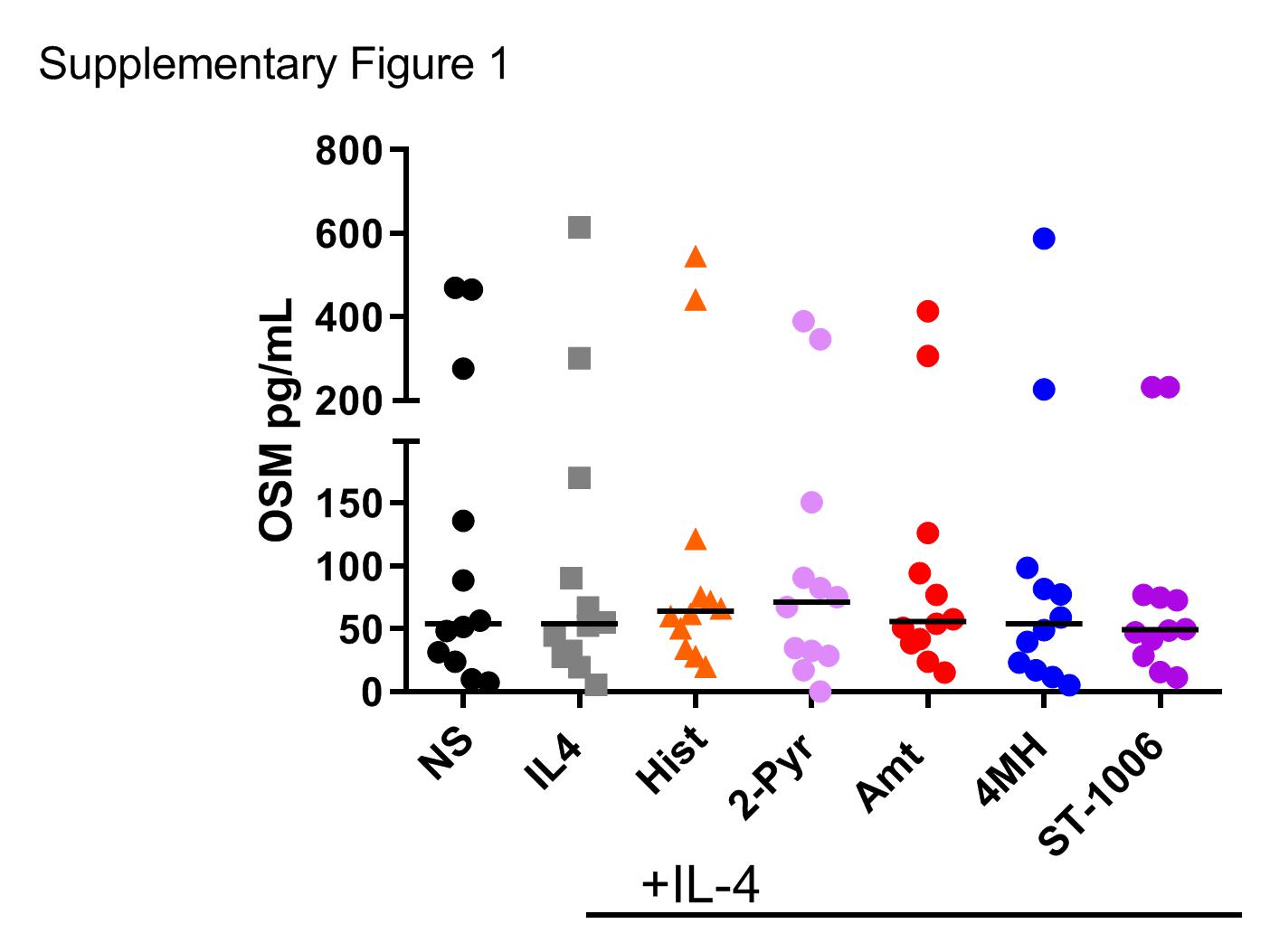

Supplement: Supplementary file 1 — Figure S1: OSM production was regulated neither by IL‐4 nor by the histamine receptor agonists in activated M2a macrophages. Primary human monocytes were obtained from PBMCs after 2‐hr adherence. M2 macrophages were differentiated from primary human monocytes in the presence of M‐CSF (10 ng/ml) for 10 days. M2 macrophages were activated by IL‐4 (20 ng/ml) for 24 hr and then stimulated with 2‐pyridylethylamine (H1R agonist), amthamine (H2R agonist), 4‐methylhistamine (4‐MH) (H2R/H4R agonist) and ST‐1006 (H4R agonist) for additional 24 hr. All histamine receptor agonists were used in the concentration of (10 μM). Secretion of OSM protein was measured by elisa technique. (n = 12 independent donors and experiments). NS, non stimulated, Hist, histamine, OSM, oncostatin M [file BPH-177-600-s001.jpg]
